# Attenuation of In Vitro and In Vivo Virulence Is Associated with Repression of Gene Expression of AIG1 Gene in *Entamoeba histolytica*

**DOI:** 10.3390/pathogens12030489

**Published:** 2023-03-21

**Authors:** Janeth Lozano-Mendoza, Fátima Ramírez-Montiel, Ángeles Rangel-Serrano, Itzel Páramo-Pérez, Claudia Leticia Mendoza-Macías, Faridi Saavedra-Salazar, Bernardo Franco, Naurú Vargas-Maya, Ghulam Jeelani, Yumiko Saito-Nakano, Fernando Anaya-Velázquez, Tomoyoshi Nozaki, Felipe Padilla-Vaca

**Affiliations:** 1 Departamento de Biología, División de Ciencias Naturales y Exactas, Universidad de Guanajuato, Guanajuato 36050, Mexico; 2 Departamento de Farmacia, División de Ciencias Naturales y Exactas, Universidad de Guanajuato, Guanajuato 36050, Mexico; 3 Graduate School of Medicine, The University of Tokyo, Tokyo 113-8654, Japan; 4 Department of Parasitology, National Institute of Infectious Diseases, Shinjuku-ku, Tokyo 162-0052, Japan

**Keywords:** *Entamoeba histolytica*, virulence, transcriptome, *EhAIG1* gene, amoeba–bacteria interaction

## Abstract

*Entamoeba histolytica* virulence results from complex host–parasite interactions implicating multiple amoebic components (e.g., Gal/GalNAc lectin, cysteine proteinases, and amoebapores) and host factors (microbiota and immune response). UG10 is a strain derived from *E. histolytica* virulent HM-1:IMSS strain that has lost its virulence in vitro and in vivo as determined by a decrease of hemolytic, cytopathic, and cytotoxic activities, increased susceptibility to human complement, and its inability to form liver abscesses in hamsters. We compared the transcriptome of nonvirulent UG10 and its parental HM-1:IMSS strain. No differences in gene expression of the classical virulence factors were observed. Genes downregulated in the UG10 trophozoites encode for proteins that belong to small GTPases, such as Rab and AIG1. Several protein-coding genes, including iron-sulfur flavoproteins and heat shock protein 70, were also upregulated in UG10. Overexpression of the *EhAIG1* gene (EHI_180390) in nonvirulent UG10 trophozoites resulted in augmented virulence in vitro and in vivo. Cocultivation of HM-1:IMSS with *E. coli* O55 bacteria cells reduced virulence in vitro, and the *EhAIG1* gene expression was downregulated. In contrast, virulence was increased in the monoxenic strain UG10, and the *EhAIG1* gene expression was upregulated. Therefore, the *EhAIG1* gene (EHI_180390) represents a novel virulence determinant in *E. histolytica*.

## 1. Introduction

*Entamoeba histolytica* is an intestinal protozoan parasite and the causative agent of amoebiasis in humans; likewise, it is one of the leading causes of death among parasitic infections in the world. An estimated 10% of the human population is infected, causing 50 million diseases, and up to 100,000 people to die each year [1,2]. While *E. histolytica* can cause invasive disease, the closely related species *Entamoeba dispar* lacks invasive potential in vivo despite being able to colonize humans [2,3].

Pathogenic amoebae use three major, well-characterized virulence factors: (i) Cell surface molecules engaged in recognition and adhesion to distinct receptors on host cells, being the Gal/GalNAc lectin that recognizes different carbohydrate components [4,5]; (ii) Several cysteine and acidic proteinases that are released by the amoebae and degrade a variety of host components, such as matrix proteins, mucins and IgA [6,7]; and (iii) Amoebapores, small protein molecules that form pores in the membranes of target cells and cause depolarization and cell death [8]. These molecules are involved in adhesion, immune evasion, colonization, and invasion, often responsible for causing disease [9,10,11,12,13]. However, these molecules cannot be completely responsible for amoebic virulence, because most of them are found in both pathogenic *E. histolytica* strains and the nonpathogenic *E. dispar* [14]. In addition to virulence factors, amoebic molecules, named determinants of virulence, participate in the expression of amoebic pathogenicity by promoting the survival of parasites under hostile conditions allowing parasites to harm the host [15,16].

To identify genes whose expression correlates with the pathogenic or virulent phenotype of *Entamoeba* species/strains, different approaches that compare the amoebic transcriptomes or proteomes of pathogenic and nonpathogenic species [17,18,19] or virulent/nonvirulent strains [20,21,22,23] have been performed. In the majority of the studies, a comparison is performed between the virulent *E. histolytica* HM-1:IMSS strain and the nonvirulent Rahman strain whose genetic backgrounds are different [13,24]. A number of genes with potential roles in stress response and virulence were downregulated in either one or both nonvirulent *Entamoeba* species/strains. These included genes encoding Fe-hydrogenase, peroxiredoxin, type A flavoprotein, and sphingomyelinase C [21,22]. However, due to the high genetic and genomic variability of *E. histolytica* isolates and strains [25,26,27], differential gene expression might be a consequence of this genetic variation rather than specific differences related to virulence.

The amoebae genes with differential expression during stress [28], host invasion [29], formation of amoebic liver abscesses [30,31], stage conversion [18], and interaction with bacteria [32,33,34] have been identified, and novel factors or determinants of virulence have been proposed. Indeed, besides some known virulence factors, several genes are upregulated during the expression of amoebic virulence, which are implicated in cellular processes, including stress response, cell signaling, vesicular trafficking [35], and antibacterial activity.

To identify additional virulence factors of this parasite, Biller and colleagues (2009), used two genetically related *E. histolytica* cell lines derived from the HM-1:IMSS strain with different pathogenic properties [36]. Cell line A produced very small lesions on the liver of experimentally infected gerbils, whereas cell line B produced considerable abscesses. In addition to decreased virulence in vivo, cell line A surprisingly revealed an increased hemolytic activity and reduced cysteine peptidase activity. In contrast, no differences between the two cell lines were found for cytopathic activity, erythrophagocytosis, digestion of erythrocytes, and resistance to complement. Nineteen genes exhibited a five-fold or higher differential expression between the cell lines. These include three RAB7 GTPases, which were found with a higher abundance in the nonpathogenic cell line A. Meanwhile, several AIG1-like GTPases were overexpressed in the pathogenic cell line B. Of the genes linked to virulence, one cysteine proteinase and one small and heavy subunit of the lectin were two-fold overexpressed in the nonpathogenic cell line A [37]. Some clones derived from pathogenic *E. histolytica* cell line B lost their ability to induce liver abscess formation in gerbils. No correlation was found between the set of in vivo and in vitro biological assays related to amoebic virulence [38].

This work describes a novel nonvirulent strain derived from *E. histolytica* HM-1:IMSS strain. This strain, termed UG10, exhibits a dramatic decrease in biological activities related to amoebic virulence, despite an identical transcriptional profile of the traditional virulence-associated genes compared with the parental virulent HM-1:IMSS strain. Remarkably, the Rab family GTPase and AIG1 family members were transcribed at higher levels in the virulent HM-1:IMSS strain than the nonvirulent UG10 trophozoites. Cultivation of *E. coli* bacterial cells with the HM-1:IMSS strain reduced its virulence and the expression of the *EhAIG1* gene, while the culture with the UG10 strain produced an increase in its virulence and the expression of this gene. Furthermore, we present evidence that the *EhAIG1* gene (EHI_180390) is a novel determinant of virulence in *E. histolytica*. Overall, the results presented here suggest that the catalog of factors/determinants of virulence in *E. histolytica* may not yet be fully elucidated.

## 2. Materials and Methods

***E. histolytica* culture.** The virulent *Entamoeba histolytica* strain HM-1:IMSS (HM-1) and the nonvirulent strains, Rahman [39] and G3 [40], were kindly provided by David Mirelman (Weizmann Institute of Science, Rehovot, Israel). All trophozoites were cultured axenically in TYI-S-33 medium supplemented with 10% adult bovine serum (Microlab Laboratories, Mexico City, Mexico) at 36 °C [41]. Trophozoites in the log phase of growth were used in all experiments. For the growth curves, 5 × 10^3^ log-phase trophozoites from axenic cultures were inoculated in 6 mL of TYI-S-33 media, and the parasite number was recorded every 24 h. Monoxenic cultures of *E. histolytica* were established as previously reported [33] with some modifications. Amoebic trophozoites were cultured with *E. coli* O55 in the presence of cefotaxime 1 μg/mL (Sigma-Aldrich, St. Louis, MO, USA) for an initial amoeba–bacteria ratio of 1:10,000.

**Generation of the avirulent UG10 cell line.** UG10 trophozoites were obtained by transfection of the parental strain HM-1 with the empty plasmid derived from pSA20 [42], which lacks the coding region corresponding to the 35-kDa subunit of the galactose/N-acetylgalactosamine-inhibitable lectin. The plasmid does not contain any gene for antisense inhibition. Transfection of trophozoites was performed by lipofection as previously described [43], and transfected trophozoites were selected with 6 μg/mL of G418 (Sigma-Aldrich, St. Louis, MO, USA). The antibiotic was gradually increased to 200 μg/mL of G418 and cultured for two years. The transfectant amoebae were subsequently cultured without G418 for three months. Loss of the plasmid was verified by the lack of PCR amplification of the *Neo* gene from total DNA isolated from trophozoites and the inability to rescue ampicillin-resistant *E. coli* transformants using this DNA. The UG10 trophozoites were further cloned by the method of limiting dilution and verified by microscopic observation [44] to establish a clonal strain. A fresh nonclonal transfectant strain (T10) was also obtained by transfection using the same empty plasmid but grown continuously with 10 μg/mL of G418.

**Induction of amoebic liver abscesses.** Six-week-old male Syrian golden hamsters (*Mesocricetus auratus*) with a weight ranging from 80 to 90 g, were used in the experiments (Institutional animal care facilities). Animals were anesthetized intraperitoneally with sodium pentobarbital, and laparotomy was performed. Animals were inoculated directly into the liver with 5 × 10^5^ *E. histolytica* trophozoites of UG10 or HM1:IMSS strains. Animals were housed and maintained with standard diet *ad libitum*. Seven days post-surgery, hamsters were anesthetized and sacrificed, and the livers were excised for lesion assessment [45]. PCR assays were used to analyze the small-subunit rRNA specific for *E. histolytica* using EH1, and EHD2 primers (Table S1) [46] were used for the liver abscess samples to corroborate the parasite infection.

**Susceptibility to complement-dependent lysis.** Assays to assess susceptibility to human complement lysis were carried out with trophozoites during the logarithmic phase of growth. A previously published protocol was followed with some modifications [47,48]. Briefly, a total of 1 × 10^6^ trophozoites were incubated in buffer (PBS, 0.5 mM MgCl_2_, 1.25 mM CaCl_2_) with 50% normal human serum for 20 min at 37 °C. As a control for amoebic viability, trophozoites were incubated with heat-inactivated normal human serum (30 min at 56 °C). Trophozoites were centrifuged at 804× *g* for 5 min, resuspended in 100 μL of PBS, and stained with 0.2% Trypan blue dye (Microlab Laboratories, Mexico) to assess cell viability. The viability of the amoebae was measured by the exclusion of Trypan blue dye. The average number of dead trophozoites that resulted from the incubation with heat-inactivated serum was subtracted from the average number of parasites killed that were incubated with normal human serum.

**Cytopathic activity on mammalian cell monolayers.** The rate of destruction of the MDCK cells (ATCC CCL-34) monolayer by *E. histolytica* trophozoites was evaluated. Amoebic trophozoites were harvested at the exponential growth phase and washed twice in PBS. MDCK cells were cultured to confluence in DMEM with 5% fetal calf serum (Gibco, Life Technologies, Waltham, MA, USA) in 24-well plates. In routine experiments, 10^5^ trophozoites from each strain were resuspended in 1 mL of TYI-S-33 without serum, added to the wells containing confluent cell monolayers, and incubated for 60 min at 37 °C. The reaction was stopped by cooling the culture plate at 4 °C for 10 min, and the wells were carefully washed twice with cold PBS. The number of mammalian cells that remained in the wells after co-incubation was determined by staining cells with methylene blue and extracting the dye as previously described [34].

**Hemolytic activity.** Hemolysis of erythrocytes (from healthy human volunteers) by intact trophozoites was performed as previously described [49]. *E. histolytica* trophozoites from each strain culture were mixed with erythrocytes in a ratio of 1:1300 in hemolysis buffer {100 mM NaCl, 30 mM KCl, 100 mM sorbitol, 0.1% bovine serum albumin, and 10 mM PIPES [piperazine-*N*,*N*9-bis(2-ethanesulfonic acid)]–Tris (pH 6.8)} and incubated for 90 min at 37 °C. The amount of hemoglobin released into the supernatant was assessed by reading the absorbance of the supernatant at 570 nm.

**Cytotoxic activity.** A simplified cell-death assay using the vital dye Hoechst 33258 (Sigma Company) was used to determine *E. histolytica* cytotoxicity [50,51]. Freshly harvested MDCK cells were mixed with trophozoites in a ratio 1000:1 in 100 μL of TYI-S-33 media without serum, centrifuged to pellet the interacting cells, and incubated at 37 °C for 1 h. The cell pellet was resuspended and treated with Hoechst 33258 (0.1% final concentration) for 5 min at room temperature and examined in an epifluorescence microscope (Zeiss Axioskop 40, Goettingen, Germany). The percentage of MDCK cells killed by amoebae was calculated by counting the fluorescent MDCK cells using the AxioVision Rel. 4.8 Software. Control MDCK cells incubated without amoebae showed a viability of >97%.

**Erythrophagocytosis and digestion assays.** Erythrophagocytosis and digestion assays were carried out as previously described [49,52]. Briefly, erythrocytes from healthy human volunteers and *E. histolytica* trophozoites from each strain were mixed in a ratio of 100:1 and incubated for 5, 15, and 30 min at 37 °C. Amoebae were chilled in an ice bath to stop erythrophagocytosis, and non-ingested erythrocytes were lysed with distilled water. Sedimented parasites were fixed with 2.5% glutaraldehyde in PBS for 30 min at room temperature. For digestion, unfixed trophozoites were additionally incubated for 2, 4, and 6 h, post-phagocytosis at 37 °C. Ingested erythrocytes were stained with benzidine, and the average number of erythrocytes per trophozoite was determined by counting using optical microscopy.

**Cysteine proteinase activity.** Proteinase activity was measured using the synthetic peptide ZArg-Arg-pNA (Bachem, Bubendorf, Switzerland) as a substrate [53]. Typically, 10^5^ trophozoites were lysed in 100 μL of lysis buffer (50 mM Tris–HCl, 10 mM NaCl, and 1% Triton X-100) (pH 7.4), and 10 μL of this lysate was combined with 90 μL of PBS and 1 μL of the 10 mM stock substrate. The release of *p*-nitroaniline was measured in a microplate reader (Multiskan Go Thermo Scientific, Waltham, MA, USA) at 405 nm.

**RNA isolation and Microarray hybridization.** RNA was isolated from 5 × 10^6^ log-phase *E. histolytica* HM-1 and UG10 trophozoites grown in 75 mL culture flasks using the Trizol reagent (Invitrogen, Waltham, MA, USA) following the manufacturer’s protocol, including a DNase I treatment (Qiagen, Venlo, Netherland). RNA was quantified by the absorbance ratio at 260 and 280 nm in the GeneQuant spectrophotometer (GE Healthcare, Chicago, IL, USA), and the integrity of isolated RNA was verified by gel electrophoresis. RNA from three biological replicates for each strain was processed using kits specified in the Affymetrix GeneChip Expression Analysis Technical Manual (P/N 702232 Rev. 3) [54]. Briefly, 5 μg of RNA was reverse transcribed to cDNA and used to synthesize biotin-labeled cRNA. After purification, cRNA was fragmented and hybridized onto a probe array chip (Eh_Eia520620F) that was custom-made by Affymetrix (Santa Clara, CA, USA) [14]. Following hybridization, arrays were washed and stained with streptavidin/phycoerythrin (Molecular Probes, Eugene, OR, USA) using an Affymetrix GeneChip Fluidics Station 450. Arrays were scanned with an Affymetrix GeneChip Scanner 3000 at 570 nm. The microarray used in this study was tailored based on information mined from the *E. histolytica* sequences deposited in the TIGR and Pathema databases. It contained 9230 probe sets for *E. histolytica* and an additional 81 control probe sets from Affymetrix.

**Analysis of microarray data.** Raw probe intensities were generated by the GeneChip Operating Software Version 1.4 (GCOS) and GeneTitan Instrument from Affymetrix. Normalized expression values for each probe set were obtained from R 2.7.0 downloaded from the BioConductor project (http://www.bioconductor.org, 10 November 2014) using robust multiarray averaging with correction for oligo sequence (gcRMA). Standard correlation coefficients were calculated using GeneSpring GX 10.0.2. The reproducibility of the experiments was determined by Pearson’s correlation coefficient and confirmed by principal component analysis. Only genes that were considered present by GCOS in at least one of three arrays for each strain were used in further analysis. One-way ANOVA analysis with Tukey’s Post Hoc test was performed to extract differentially expressed genes. The *p*-values were calculated using Welch’s test and were corrected by Benjamini–Hochberg method. Microarray data are available at the ArrayExpress database hosted at www.ebi.ac.uk/arrayexpress (accessed on 20 January 2023), under the accession number E-MTAB-3525.

**Plasmid construction and production of *E. histolytica* transfectant overexpressing *EhAIG1* gene.** Standard cloning techniques previously described [55], were used for routine DNA manipulation, subcloning, and plasmid construction. DNA encoding the open reading frame of amoebic *EhAIG1* gene (EHI_180390) was generated by PCR from *E. histolytica* HM-1 DNA using High Fidelity DNA Polymerase (HiFi polymerase Platinum, Invitrogen). *EhAIG1* coding region (1008 bp) was amplified using the forward and reversed primers containing *Bgl* II and *Xho* I restriction sites (Table S1), respectively. PCR product was cloned into pGEM-T easy (Novagen) and subcloned into the expression vector pEhEx [56] to produce pAIG1 plasmid construction. The plasmid was sequenced and used to transfect the UG10 strain trophozoites. Transfected cells were selected as described above to obtain the UG10spAIG1 transfectant. Trophozoites were transfected with the empty plasmid (UG10pEhEx) as a control. The amount of G418 was gradually increased to 36 μg/mL for both transfectants and used for all the experiments.

**Quantitative real-time PCR.** Total RNA was isolated from trophozoites using the Aurum Total RNA Mini Kit (BIO-RAD), including DNAase I treatment as directed by the manufacturer. For the synthesis of first strand cDNA, 3 μg of total RNA (DNA-free) isolated from amoebic trophozoites was reverse transcribed using Oligo (dT) and reverse transcriptase from the SuperScript II RT-system (Invitrogen) according to the manufacturer’s instructions. For quantitative real-time PCR experiments, sense and antisense primers were designed to amplify approximately 150 base pairs of the target gene sequences (Table S2). qPCR was performed using the Step One Real-Time PCR System (Applied Biosystems, Waltham, MA, USA) and Fast SYBR Green Master Mix (Applied Biosystems) following the manufacturer’s protocol.

**Statistical Analysis.** Data generated were analyzed using an analysis of variance (ANOVA) to examine the effect of gene overexpression or bacteria interaction on cell monolayer destruction and hemolytic activity. The means were compared using the Tukey–Kramer test to identify statistically significant differences between them. The statistical analysis was performed with the aid of R (http://r-project.org/ (accessed on 20 January 2023) Version 4.0.2) and JMT Pro13 (jmp.com (accessed on 20 January 2023)) statistical software.

Ethics statement. Animal handling of Syrian golden hamsters was conducted according to ethical guidelines of care and use of laboratory animals. A bioethics committee (formerly Centro de Investigaciones en Bioética de la Universidad de Guanajuato, at present Comité Institucional de Bioética en la Investigación de la Universidad de Guanajuato) approved the document including all experimental procedures related to the use of experimental animals or human serum and erythrocytes. The date of the approval document was February 24, 2014. No identification number was assigned. All procedures of the research by using experimental animals were performed according to the national regulation (Norma Oficial Mexicana NOM-062-ZOO-1999, Especificaciones técnicas para la producción, cuidado y uso de los animales de laboratorio). Experimental procedures with human serum and erythrocytes were performed according to the National Regulation of Scientific Research in human beings (Reglamento de la Ley General de Salud en material de investigación para la salud, 2014).

## 3. Results

**Figure 1:**
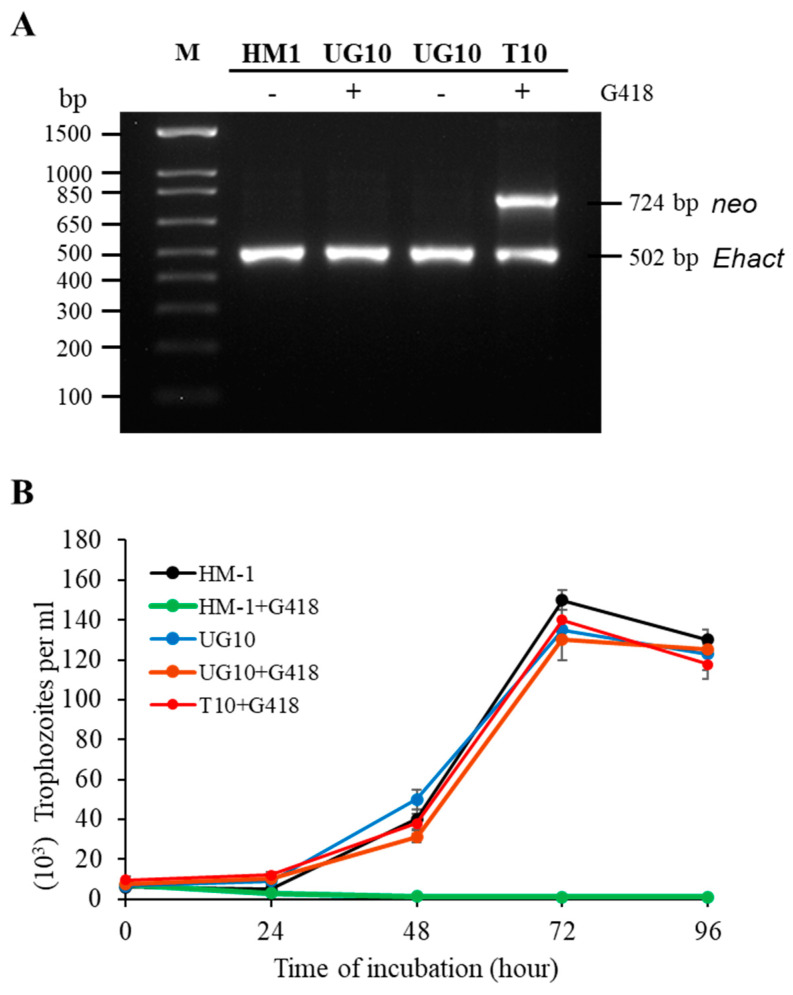
*E. histolytica* UG10 strain is resistant to G418 despite plasmid loss. (**A**). UG10 strain was negative for *neo*-marker amplification by PCR in the presence of G418. +, amoebae were grown with 10 μg/mL of G418; −, amoebae were grown without G418. PCR was performed using specific oligonucleotides (Table S1), and PCR products were run on an agarose gel. M, DNA ladder. (**B**). Seed-cultures of 5000 log-phase trophozoites from the UG10 and HM-1 strains were grown with (10 μg/mL) and without G418 at 36 °C and counted every 24 h. The T10 transfectant strain was grown with G418. Growth curves were performed three consecutive times in duplicates. The standard error is indicated.

***E. histolytica* UG10 strain is resistant to G418.** UG10 was a derivative of the highly virulent HM-1 strain. It was obtained by transfecting HM-1 with an empty pAS20 plasmid containing a *neo* cassette and selected with 200 μg/mL of G418. The plasmid was eventually lost by growing the amoebae without G418 for three months, which was confirmed by PCR (Figure 1A), and the lack of plasmid rescue in *E. coli* cells. A single cell-originated clonal strain UG10 was obtained as described in the Materials and Methods Section. T10 is a nonclonal line transformed with the same empty pAS20 plasmid and maintained with 10 μg/mL of G418, and retains the plasmid, confirmed by PCR (Figure 1A). Although UG10 cells lost the plasmid, they were able to grow with 10 μg/mL of G418 at a similar rate to the parental HM-1 strain grown in the absence of antibiotics (Figure 1B). The IC_50_ values for HM-1 and UG10 were 1.94 and 12.34 μg/mL of G418, respectively.

**The UG10 strain displays a nonvirulent phenotype.** While mechanisms for G418 resistance are still unknown, the in vivo virulence phenotype of UG10 was examined and compared to the parental HM-1 strain. In addition to HM-1 and UG10, we also used the nonvirulent G3 and Rahman strains. 5 × 10^5^ trophozoites were inoculated to produce amoebic liver abscesses in hamsters. Trophozoites of the HM-1 strain produced large abscesses that occupied approximately 80% of the whole liver, whereas UG10, similarly to Rahman and G3, does not produce visible lesions in the liver (Table 1), even with an inoculum of 3 × 10^6^ trophozoites. Cytopathic, cytotoxic, and hemolytic activities in vitro were also examined. UG10 trophozoites exhibited a dramatic 80–85% decrease in these activities compared to the HM-1 strain. UG10 trophozoites had very low cytotoxic, hemolytic, and cytopathic activities and were most sensitive to human complement (Table 1). This UG10 avirulent phenotype was not affected by the presence or absence of G418 in the culture medium (Figure S1). These results indicate that UG10 trophozoites exhibit markedly reduced virulence-associated activities. Importantly, this set of in vivo and in vitro biological assays was evaluated periodically without significant changes over the years, indicating that all the strains used have a stable phenotype under the same growth conditions. HM-1 exhibited the highest cytopathic, cytotoxic, and hemolytic activities, complement resistance, and liver abscesses formation in hamsters (Table 1), consistent with previous reports. No remarkable differences between the strains were found for erythrophagocytosis and digestion of erythrocytes, except Rahman, which is known for having lower phagocytic activity [57]. The cysteine protease activity remained unchanged among the strains, except for the G3 strain, which showed higher proteolytic activity (Table 1).

**Figure 2:**
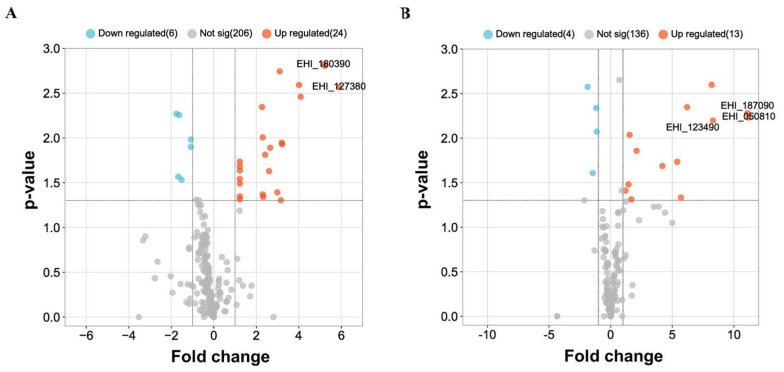
Significance of expression analysis. Volcano plots were used to identify the most significant upregulated genes in the analysis between upregulated genes when comparing HM1:IMSS vs. UG10 and vice versa. Panel (**A**): Volcano plot comparing HM1:IMSS vs. UG10 expression levels. Panel (**B**): Volcano plot comparing UG10 vs. HM1:IMSS. Genes evaluated for characterization are indicated. Plots were generated in R Studio (RStudio Team (2020). RStudio: Integrated Development for R. RStudio, PBC, Boston, MA URL http://www.rstudio.com/ (accessed on 20 January 2023) Version 4.0.2.

**Comparison of transcriptomic profiles between the nonvirulent UG10 strain and its parental HM-1 strain.** In order to elucidate the underlying mechanisms for the decrease in virulence in UG10, we investigated the differential gene expression between UG10 and its parental strain HM-1. We performed an analysis of global gene expression analysis using a custom-made Affymetrix microarray representing 9311 genes of *E. histolytica*. A heatmap analysis of the downregulated comparison of the up and downregulated genes between HM1:IMSS and UG10 strains is demonstrated (Figure S1). We identified 106 (1.138%) and 51 (0.547%) genes that were differentially expressed at least two and five-fold, respectively. Out of the 106 genes, 51 genes were downregulated, and 55 genes were upregulated (Tables S3 and S4). Volcano plots were used to identify the most significant upregulated genes in the analysis between upregulated genes when comparing HM1:IMSS vs UG10 and vice versa (Figure 2). Out of the 51 downregulated genes, 12 genes (23.5%) were assigned with putative biological functions, namely signaling, oxidation and reduction chemistry, lipid and carbohydrate degradation, nucleic acid interaction, stress response, and protein transport (Table 2). The remaining 39 genes (76.5%) were categorized into genes encoding hypothetical proteins (Table S3). Out of the 55 upregulated genes, 14 (25.5%) were assigned putative biological functions associated with bacterial interaction/killing, signaling, nucleic acid interaction, lipid and carbohydrate degradation, and cell metabolism (Table 3). The remaining 41 genes (74.5%) were categorized as genes encoding hypothetical proteins (Table S4). Remarkably, there was no difference in the expression of genes directly linked to virulence, such as amoebapore (EHI_159480), Gal-lectin (EHI_035690, EHI_148790, EHI_065330), and cysteine proteinase (EHI_168240). This observation was confirmed by qRT-PCR that the relative expression of such genes does not significantly change (Table S5). Altogether these results suggest that additional factors or determinants of virulence may cause the observed difference in phenotype between HM-1 and UG10, such that the catalog of genes contributing to amoebic virulence still is not yet complete.

**Expression of putative virulence-associated genes in *E. histolytica* strains of different virulence phenotype.** In order to verify the correlation between up or downregulation of the identified genes and the virulence phenotype, we selected two representative genes that were significantly upregulated [AIG1 (EHI_180390) and EhRab8B (EHI_127380)], and two significantly downregulated genes [WH2 motif domain-containing protein (EHI_050810) and Heat shock protein 70 (EHI_123490)] in the virulent HM-1 strain compared to UG10 strain. Transcription of these genes in HM-1, G3, UG10, and Rahman strains, whose apparent virulence in vitro and in vivo was demonstrated above, was examined using qRT-PCR. The expression of these genes in HM-1 and UG10 is in good agreement with the trends seen by DNA microarray analysis. *EhAIG1* (EHI_180390) showed a correlation between the expression levels and virulence strains (Table 4).

**Figure 3:**
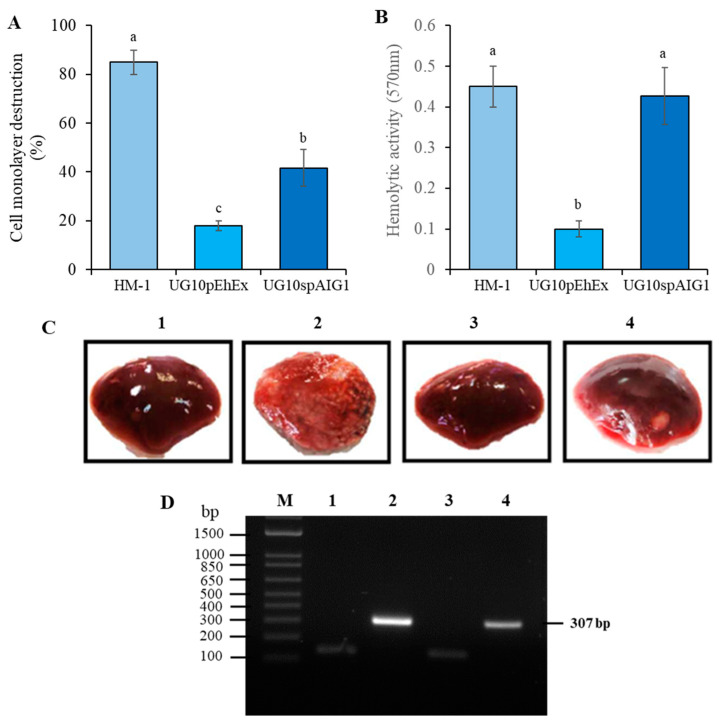
Virulence of UG10 strain overexpressing the *EhAIG1* EHI_180390 gene. UG10 nonvirulent mutant transfected with the empty plasmid pEhEx (UG10pEhEx) and overexpressing the EHI_180390 gene (UG10spAIG1) were grown with 36 μg/mL of G418. (**A**). Cytopathic activity. The rate of destruction of MDCK cells monolayer was determined after incubation with 1 × 10^5^ trophozoites for 60 min at 37 °C. (**B**). Hemolytic activity. Hemolysis of human erythrocytes by intact trophozoites was determined by spectrophotometric quantification of hemoglobin released after 90 min of interaction at 37 °C. Three independent experiments were performed in triplicate for each in vitro biological assay. The bars represent the mean number ± SE. Different letters over the bars represent statistically significant differences at *p* ≤ 0.05 (Tukey–Kramer test). (**C**). Induction of amoebic liver abscesses in hamsters. Animals were inoculated intrahepatically with 1 × 10^6^ and 2 × 10^6^ trophozoites for HM-1 and transfectants, respectively. (**D**). PCR amplification of amoebic ribosomal gene directly from liver abscesses or from liver without abscesses (M, DNA ladder; 1, Negative control; 2, HM-1; 3, UG10pEhEx; 4, UG10spAIG1).

**Overexpression of *EhAIG1* (EHI_180390) gene and its role in *E. histolytica* virulence.** To investigate whether *EhAIG1* (EHI_180390) gene plays a role in virulence, a transfectant line that overexpresses *EhAIG1* (EHI_180390) was generated from nonvirulent UG10 (UG10spAIG1). Approximately seven-fold overexpression of the transcript was observed in the transformant by qRT-PCR. UG10spAIG1 cells showed a three-fold increase in cytopathic activity compared to the control (UG10pEhEx), and thus partially (40%) rescued the cytopathic activity of the parental HM-1 strain (Figure 3A). UG10spAIG1 cells exhibited a 4.5-fold increase in hemolytic activity, which was comparable to the level of activity in HM-1 (Figure 3B). UG10 and UG10pEhEx strains exhibited similar in vitro virulence activities. We also examined the ability of the UG10spAIG1 transfectant to induce the formation of liver abscesses in hamsters. The UG10spAIG1 trophozoites only marginally recovered the ability to produce liver abscesses, whereas UG10pEhEx did not (Figure 3C). Live parasites in the liver abscesses were confirmed by further 48-h cultivation of the excised abscesses in TYI-S-33 medium followed by the microscopic demonstration of motile trophozoites and PCR amplification of 18S rRNA genes (Figure 3D). Taken together, the virulence phenotype seen in the parental HM-1 was partially regained by overexpression of a single *EhAIG1* (EHI_180390) gene.

**Figure 4:**
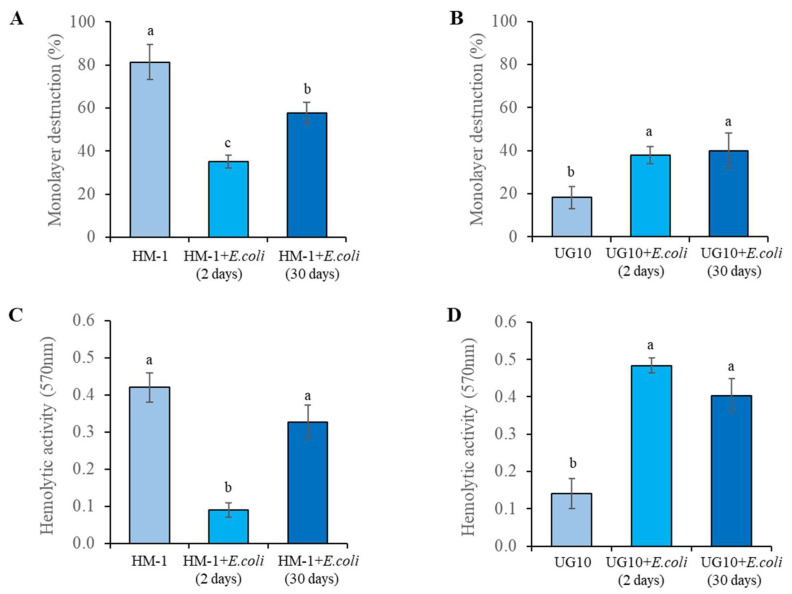
*E. histolytica* virulence and *EhAIG1* (EHI_180390) gene expression in response to *Escherichia coli* O55. Cytopathic activity. The rate of destruction of MDCK cells monolayer was determined after incubation with 1 × 10^5^ trophozoites of HM-1 (**A**) and UG10 (**B**) strains co-cultured with *E. coli* O55 for two and 30 days for 60 min at 37 °C. Hemolytic activity. Hemolysis of human erythrocytes by intact trophozoites of HM-1 (**C**) and UG10 (**D**) strains co-cultured with *E. coli* O55 for two and 30 days was determined by spectrophotometric quantification of hemoglobin released after 90 min of interaction at 37 °C. The results show the average of three independent experiments in duplicate. The bars represent the mean number ± SE. Different letters over the bars represent statistically significant differences at *p* ≤ 0.05 (Tukey–Kramer test).

***E. histolytica* virulence and *EhAIG1* (EHI_180390) gene expression in response to *Escherichia coli* O55.** Two day monoxenic culture of trophozoites of HM-1 with *E. coli* O55 under an amoeba–bacteria ratio (1:10,000) showed a decrease of cytopathic and hemolytic activity in a similar way to that previously reported [32,33], and after one month of monoxenic culture, the trophozoites recovered 80% of their cytopathic and hemolytic activity (Figure 4A,C). Under the same conditions of monoxenic culture, the avirulent UG10 trophozoites increased their cytopathic and hemolytic activity (Figure 4B,D). The *EhAIG1* (EHI_180390) gene expression in the HM-1 strain co-cultured with bacteria for two days was downregulated (~two-fold) and was recovered after 30 days of monoxenic culture. The same gene in the UG10 strain was upregulated 2.4-fold and 5.1-fold after two and 30 days of monoxenic culture, respectively (Table 5). The AIG1 EHI_180390 gene is differentially expressed in trophozoites co-cultured with *E. coli* O55, which correlates with bacteria-induced virulence changes in vitro. After 30 days of monoxenic culture, the same level of expression gave the same level of monolayer destruction/hemolytic activity. Interestingly, the effect of *E. coli* O55 bacteria cells on the virulence of the HM-1 and UG10 strains is the opposite, suggesting that they may also differ in their regulation.

## 4. Discussion

Few studies show attenuation of virulence in isogenic *E. histolytica* strains to date. Biller et al. analyzed two isogenic lines, A and B, derived from the HM-1:IMSS strain. No tight association was found between in vitro activities related to amoebic virulence and the ability to produce liver abscesses. Transcriptomic comparisons of the A and B cell lines showed differences in virulence-related genes (peptidases and lectins) and Rab and AIG1 genes [36,37]. The present work is the first report of a nonvirulent *E. histolytica* strain derived from the virulent HM-1 strain, which markedly exhibits reduced biological activity related to virulence which is not associated with the transcript levels of known virulence factors. Furthermore, this study revealed a number of putative genes differentially regulated in the virulent HM-1 and the nonvirulent UG10 strain. This prompts the question of the role of such genes in *E. histolytica* physiology and their involvement in the pathogenesis process.

*E. histolytica* isolates present a large number of genotypes with a high level of genetic and genomic diversity, and some are associated with increased virulence in humans [2,25]. However, there is no clear correlation between *E. histolytica* genotypes and amoebic virulence or symptomatology [2]. Due to the genetic variability among *E. histolytica* strains and isolates, virulence phenotype differences between strains or isolates might reflect inter-isolate/strains or even environmental variations rather than specific differences linked to virulence.

In the present work, an attenuated strain UG10 was created in an unprecedented protocol from the highly virulent HM-1 strain: transfection with a plasmid, selection with G418 resistance, and removal of drug selection resulting in plasmid loss. The UG10 trophozoites showed a dramatic decrease in cytopathic, cytotoxic, and hemolytic activity; they were also sensitive to human complement and were unable to produce liver abscesses even at the highest tested inoculum dose. Strikingly, expression levels of known virulence factors such as Gal/GalNAc specific lectin, cysteine proteases, and amoebapore remained similar between HM-1 and UG10 strains. The relative virulence of different strains of *E. histolytica* has been shown to change due to variations in in vitro cultivation [33,41,58,59].

*E. histolytica* HM-1 strain produced considerable liver abscesses, low sensitivity to human complement, and high cell-damaging activities, while G3, Rahman, and UG10 were unable to produce any liver abscesses and exhibited increased sensitivity to human complement and low cell-damaging activities. Phagocytosis was reduced only in the Rahman strain as reported previously [57]. The virulence of *E. histolytica* strains was not correlated to cysteine proteinase activity as previously reported [60]. We compared the transcriptomes of UG10 and its parental HM-1 trophozoites. In total, 106 (1.138%) and 51 (0.547%) genes were differentially expressed at least two and five-fold, respectively. Gene expression of proteins involved in virulence remained unchanged between virulent and nonvirulent strains, which agreed well with the qRT-PCR data (Table S5). Most differentially expressed genes belonged to the family of small GTPases. These are of special interest as they are putative *E. histolytica* virulence determinants. Rab and AIG1-GTPases were more highly expressed in the virulent HM-1 strain than in avirulent UG10.

Upregulation of four AIG1 family protein members in the virulent HM-1 strain ranged from six to 30-fold. Three of them (EHI_180390, EHI_022500, EHI_115160) were also more highly expressed in the pathogenic cell line B of *E. histolytica* than in the nonpathogenic cell line A [37]. Previous studies have shown that *EhAIG1* genes are overexpressed in virulent HM-1 as compared to the avirulent Rahman strain [20] and also overexpressed in HM-1 trophozoites obtained from a murine model of amoebic colitis [29].

The *E. histolytica* AIG1 family members show structural similarities to members of the GTPases of the immunity-associated protein (GIMAPS) family and to the immunity-associated nucleotide-binding protein (IAN) family of AIG1-like GTPases, which are conserved between vertebrates and angiosperm plants [61].

**Figure 5:**
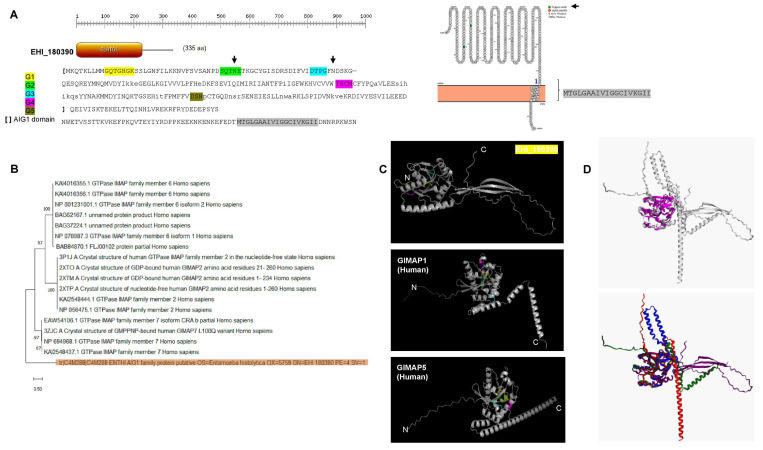
Sequence and structural features of the AIG1 EHI_180390. (**A**)**.** Prosite scan and Protter analysis of EHI_180390. The main motifs are indicated on the left of the image, the sequence represents the hits with AIG1 consensus. A predicted membrane-associated sequence located at the C-terminal region is found. Two putative glycosylation sites are indicated with black arrows. (**B**). Phylogenetic analysis of the best BLASTp hits when comparing EHI_180390. In shade, the EIH_180390 is indicated. Analysis was conducted with MEGA with 500 bootstrap iterations. (**C**). Protein model generated with AlphaFold2 and its comparison with experimentally determined human GIMAP homologs GIMAP 1 (AlphaFold database accession number AF-Q8WWP7-F1) and GIMAP5 (AlphaFold2 accession number AF-Q96F15-F1). The signature motifs are indicated with the same color scheme as in Panel A, and N and C indicate the N-terminal and C-terminal ends of each protein. Panel (**D**)**.** Structural comparison between EHI_180390 with GIMAP7 (PDB 3ZJC), AlphaFold models shown in Panel C, common core is indicated in magenta (upper image), and each protein position is indicated in the lower image. In blue, GIMAP7, in green GIMAP1, in red GIMAP5, and in purple EHI_180390.

At least 47 genes are predicted to encode AIG1 proteins in the *E. histolytica* genome. The *E. histolytica* AIG1 family members of 20–45 kDa have the first three of the five GTP-binding sites conserved in other organisms [37,61], and also conserved in *E. invadens*, *E. dispar,* and *E. moshkovskii* [37], which are present in the AIG1 gene analyzed in this study (Figure 5A). The protein has a predicted C-terminal transmembrane anchoring motif (Figure 5A) that may be involved in extravesicular and extracellular activity. Furthermore, the folding comparison suggests that this AIG1 has a conserved catalytic domain, but the rest of the protein lacks the canonical alpha helix shown in GIMAP proteins from humans (Figure 5C, and for overall comparison, Figure 5D). The AIG1 (avrRpt2-induced gene) family of GTPases has a distinctive AIG1 domain and has a peculiar phylogenetic distribution (for this AIG1 see Figure 5B) and an uncertain functional role [58,59]. These characteristics are present in AIG1 EHI_180390 which is consistent with its functional role as a small GTPase protein.

AIG family members have been described as virulent-related genes and are recurrently found in differential gene expression analyses. AIG1 family genes have frequently been found to be differentially expressed in different strains [18,36,62], species [62,63,64], clinical isolates [23,65], life-cycle stages [18], and host–parasite interactions [29,63,66].

The regulation of some members of the AIG family is contrasting since sometimes it can be associated with a virulent phenotype, while in other conditions with a nonvirulent one. These intriguing features lead us to further explore the role of AIG genes and elucidate their role in the pathogenesis of *E. histolytica*. Furthermore, due to belonging to a large family of genes, individual members could express themselves in a specific way following particular stimuli, including amoeba–bacteria interaction.

Overexpression of the EHI_180390 gene in the nonvirulent UG10 strain described in this work resulted in augmented in vitro cell-damaging activities and partially regained liver abscesses production. The EHI_180390 gene reported in this study was not differentially expressed in the stool samples from symptomatic and asymptomatic patients [67]. The *EhAIG1* (EHI_176590) gene was detected in 56% of stool samples from symptomatic patients infected with *E. histolytica*, but only in 15% of stool samples from asymptomatic individuals [67]. Overexpression of the EHI_176590 gene in strain HM-1:IMSS cl6 resulted in increased formation of cell-surface protrusions and enhanced adhesion to human erythrocytes, contrasting with the results of overexpression or gene silencing of EHI_176590 which exhibited reduced and increased liver abscess formation, respectively, showing an opposite effect on liver abscess formation and virulence hallmarks in vitro. The EHI_176590 gene was not differentially expressed in UG10 and HM-1 strains from our work. The pathogenesis of the clinical strains is related to gene expression patterns rather than the expression of individual genes [23]. However, the AIG1 expression (EHI_180390) was higher in the hypervirulent Ax19 strain (over seven-fold), in comparison with Ax22 and Ax11 strains [23]. This indicates that the expression of EHI_180390 is in good agreement with the data shown in this work. All these suggest that the AIG1 genes may have non-redundant roles in virulence that depend on the genetic background of the parasite and the environmental conditions imposed by the host.

The first family member, AIG1, was discovered in *Arabidopsis thaliana,* and its expression was induced in defense against bacterial infection or abiotic stressors [68]. Many different bacterial communities populate the large intestine of humans, and *E. histolytica* grows in their midst, where the bacteria are an important nutritional source. Bacterial populations associated with *E. histolytica* have been identified in the gut environment, such as *Lactobacillus ruminus* [69] and the family of Enterobacteriaceae [70]. It has been shown that the bacterial microbiota is important for *E. histolytica* to survive oxidative stress and to establish itself in the intestinal mucosa to colonize and potentially invade it [70]. The bacterial microbiome produces metabolites important for the parasite to grow [18]. The gut microbiota may significantly influence the host’s immune response and/or *E. histolytica*’s virulence [13,71]. The interaction with microbiota-amoeba-mucus could be critical in the onset and progression of disease [72]. The feeds on bacteria and their pathogenicity have been directly linked to exposure to bacterial microbiota [73]. The bacterium-parasite interaction is very selective because only bacteria with the appropriate recognition molecules are ingested by *E. histolytica* [34,74]. Several studies have shown that the interaction between specific bacteria and *E. histolytica* plays an important role in the growth and virulence of the parasite [32,33,75,76].

Factors from *E. histolytica* that may have a role in signaling triggered by the interaction between trophozoites and bacteria are the immunity-associated GTPases, similar to the AIG proteins involved in the defense mechanisms of plants and invertebrates against bacteria [37,77]. In the gastropod *Biomphalaria glabrata* [77], four AG1s are upregulated following exposure to LPS and peptidoglycan [78]. It has been suggested that AIG-GTPases have an important role in the amoeba–bacteria interaction [79]. Our results support the close relationship of the AIG1 genes with the virulence of *E. histolytica*. However, there is little evidence of the relationship between AIG1, amoebic virulence, and bacteria interaction. The expression of the AIG1 EHI_180390 gene, besides showing a close correlation with the virulence of *E. histolytica*, is differentially expressed in trophozoites co-cultured with *E. coli* O55, which correlates with bacteria-induced virulence changes in vitro. Surprisingly, the effect of *E. coli* O55 bacteria cells on the virulence of the HM-1 and UG10 strains is the opposite. The mechanism of this interaction and the responsible virulence molecules remain to be elucidated.

The AIG1 homolog EHI_180390 was further analyzed for sequence and structural features. A prosite scan revealed conserved signatures of the AIG1 family, and Protter and Topcons topology confirmed that this protein contains a single membrane-anchoring sequence (Figure 4A). In the search for homologs to human proteins, BLASTp and subsequent phylogenetic reconstruction of obtained hits suggested that EHI_180390 is a distant homolog of human GIMAP6, 2, and 7 (Figure 4B). Intriguingly, GIMAP1 and 5 contain a single C-terminal transmembrane helix that anchors them to the Golgy and lysosomal membranes [80]. Further analysis using a high-quality protein model of EHI_180390 revealed a C-terminal disordered region (Figure 4C) where the C-terminal helix should be placed. By comparing the AlphaFold2 models of human GIMAP1 and 5, the signatures of the AIG1 family are in relatively similar positions. However, the C-terminal region is highly divergent (for comparison, Figure 4D shows the structural alignment including GIMAP 2, which has also been shown to be associated with lipid droplets [81,82]. The divergent C-terminal region suggests that in this parasite, EHI_180390 may interact with other proteins in response to specific cell compartments or signals and thus result in a particular cell function as previously shown for GIMAP6 and its interaction with GABARALP2 to regulate its abundance and function in autophagy [80].

All the efforts to assess the homology of EHI_180390 with other proteins render unfruitful results. The phylogenetic reconstruction suggests that, as previously suggested, many proteins in *E. histolytica* have ancestry with human and other animal proteins [46], in this case, EHI_180390 may be the ancestral protein for membrane-anchored AIG1 proteins (Figure 4B). The long C-terminal extensions found in EHI_180390 and the close homologs shown in Figure 4B suggest that, as shown previously for GIMAP2 and GIMAP7, they are needed to interact to hydrolyze GTP [82]. Furthermore, to form scaffolds as shown for GIMAP2 and GIMAP5, their binding to the membrane restricts mobility but in turn, forms scaffolds in the GTP-bound form [83]. The three antiparallel beta strands shown in the C-terminal region of EHI_180390 suggest that they might play a role in forming scaffolds with other proteins as discussed previously.

The lack of homology to a known receptor or protein with a defined role besides the AIG1-family proteins in *E. histolytica* reduces the possibility of drawing a direct role in either sensing or initiating a regulatory cascade. As shown in Figure 4, the membrane anchoring domain is also highly conserved in these proteins (when present), and the homology between them and the conserved catalytic motifs in the majority of these proteins, suggests that they are highly conserved in *E. histolytica* (Supplementary Figure S2B,C). However, these proteins’ homology and structural features, specifically those found to be differentially regulated in two contrasting phenotypes, suggest that EHI_180390 might be involved in cell migration. Specifically, GIMAP7 has been demonstrated to be involved in immune cell infiltration [84]. The membrane anchoring motif found in EHI_180390 suggests that this protein may be involved in either trafficking virulence factors to be secreted or the binding in the surface to either bacteria or host cells, acting as a GTP-induced switch. In future studies, the subcellular location of AIG1-family proteins found to be differentially regulated will be determined to draw meaningful conclusions regarding their role. However, with the evidence shown here, the high number of genes encoding AIG1 proteins strongly suggests many are non-redundant.

A subset of cell signaling-related genes, including Rab family GTPases, the Ras, and the Rho family, were also identified as differentially expressed genes. Out of 25 genes that were upregulated more than two-fold in the virulent HM-1, nine belong to the small GTPase superfamily, Rab, Ras, and Ran. Four members of Rab genes were overexpressed 20–50-fold in the virulent HM-1.

Interestingly, these four Rab genes (8A, 8B, F1, and X13) identified in the present study had not been upregulated in virulent B clone in the previous study [34]. Their expression is probably important for in vivo virulence. Only the EhRab8A gene has been involved in phagocytosis in *E. histolytica* [85]. In contrast, the same Rab gene (EhRab7G) upregulated in UG10 (EHI_187090) was also upregulated in the nonvirulent line A. This was the unique Rab gene associated with the nonvirulent genotype in both studies. Although both studies use the HM-1 reference strain and their liver-passaged and laboratory-attenuated derivatives with the same genetic background, no key gene was identified to determine the gain or loss of virulence.

Rab proteins are essential molecular switches regulating the fusion of vesicles with target membranes through the conformational change between active (GTP-bound) and inactive (GDP-bound) forms [86,87,88]. The *E. histolytica* genome encodes 102 Rab family G proteins suggesting an unusually high degree of the complex regulatory network controlling vesicular trafficking in trophozoites [89,90]. EhRabA, B, 5, 7A, 7B, 8A, 11A, and 11B have been studied in *E. histolytica*, evidencing roles in phagocytosis, transport, and localization of virulence factors [56,90,91,92,93,94,95]. The role of Rab proteins differentially regulated in highly virulent or avirulent strains remains elusive.

WH2 motif domain-containing protein (Gene EHI_050810) was upregulated in UG10 compared to HM-1. The WH2 domain is a 35 residues actin monomer-binding motif found in different actin cytoskeleton regulators [96]. The *E. histolytica* genome revealed seven genes encoding WH2 motif-containing proteins. This is the first report showing differential expression of WH2 motif-containing proteins. Gene EHI_050810 also contains a nucleoporin complex domain involved in endocytosis, sorting, and trafficking regulation within the cell.

Interestingly, four iron-sulfur flavoprotein genes were upregulated in the nonvirulent UG10. Iron-sulfur flavoproteins constitute a family of redox-active proteins found predominantly in anaerobic prokaryotes [97] and eukaryotes. Proteins in this family usually contain a flavin mononucleotide (FMN) cofactor and iron-sulfur [Fe-S] clusters with a compact cysteine motif. There are at least seven genes for iron-sulfur flavoproteins in the genome of *E. histolytica* [98]. The expression of iron-sulfur flavoproteins is regulated by reactive oxygen species, in agreement with their proposed function in bacterial anaerobes to cope with reactive oxygen species by reducing O_2_ and H_2_O_2_ to water [97]. In addition to oxidative stress, iron-sulfur flavoproteins, and related proteins were also induced by the deprivation of sulfate or L-cysteine in bacteria [99]. In *E. histolytica* trophozoites, five of these genes were upregulated ≥ 3-fold upon L-cysteine deprivation [14]. The upregulated expression of iron-sulfur flavoprotein genes in UG10 trophozoites could be related to an alteration in cysteine metabolism or stress oxidative response pathways.

Four upregulated genes encoding hypothetical proteins were the most differentially expressed transcripts in the nonvirulent UG10 strain. Among the top ten upregulated genes in each strain, eight genes upregulated in the nonvirulent UG-10 and five upregulated in the virulent HM-1 correspond to hypothetical proteins (Tables S3 and S4). These results suggest that virulence in *E. histolytica* may involve many more genes than anticipated, with complex roles yet to be elucidated. Although we demonstrate for the first time that the *EhAIG1* (EHI_180390) gene participates in *E. histolytica* virulence, its precise role in the regulation and the mechanisms of its actions remain to be determined.

## Figures and Tables

**Table 1:** Virulence phenotype characterization of *Entamoeba histolytica* strains.

Biological Activities	HM-1	G3	UG10	Rahman
Production of amoebic abscess ^a^	Yes (10/10)	No (0/5)	No (0/10)	No (0/5)
Susceptibility to complement ^b^ Cytopathic effect ^c^	47.0 ± 11 87.6 ± 10	28.5 ± 7 * 25.0 ± 3 *	19 ± 6 * 15 ± 4 *	23 ± 12 * 15 ± 4 *
Cytotoxic effect ^d^	42.0 ± 10	32.0 ± 5 *	7 ± 1 *	12 ± 2 *
Hemolytic activity ^e^	0.4 ± 0.06	0.25 ± 0.02 *	0.08 ± 0.02 *	0.10 ± 0.02 *
Erythrophagocytosis ^f^ 5 min	5.4 ± 1.2	5.7 ± 1.1	4.4 ± 0.4	2.1 ± 0.3 *
15 min	7.5 ± 1.1	8.0 ± 1.0	6.1 ± 0.5	2.9 ± 4.5 *
30 min	10.3 ± 1.0	9.6 ± 0.5	10.4 ± 1.5	4.5 ± 0.5 *
Digestion of human erythrocytes ^g^ 0 h	9.00 ± 0.6	9.4 ± 0.8	8.8 ± 0.5	5.4 ± 0.84 *
2 h	5.00 ± 0.4	6.8 ± 0.4	6.2 ± 0.3	3.2 ± 0.59 *
4 h	1.32 ± 0.5	2.7 ± 0.6	2.2 ± 0.3	0.6 ± 0.20 *
6 h	0.45 ± 0.3	1.0 ± 0.5	0.8 ± 0.2	0.3 ± 0.05
Cysteine protease activity (nmol/min/mg) ^h^	328 ± 46	589 ± 75 *	386 ± 21	306 ± 16

^a^ Yes: HM-1 induces liver abscesses in all the hamsters inoculated, affecting 80–90% of hepatic lobe inoculated with 5 × 10^5^ trophozoites. No: None of the strains (G3, UG10, or Rahman) induced liver abscesses. ^b^ 1 × 10^6^ trophozoites were incubated with normal human serum for 20 min at 37 °C. Data are shown as a percentage of viable parasites. ^c^ % of the destruction of MDCK cell monolayer after 60 min of incubation. ^d^ % of MDCK cells killed by amoebae was determined by counting cells stained with Hoechst 33258. ^e^ Hemoglobin released was assessed by reading the supernatant at 570 nm. ^f^ Number of erythrocytes phagocytosed per trophozoite. ^g^ After 30 min of interaction, unphagocytosed erythrocytes were lysed, and the remaining erythrocytes per trophozoite were counted. ^h^ Z-arg-Arg-pNA was used as a substrate. * Indicates *p* < 0.05 in comparison with HM-1 strain.

**Table 2:** Subset of genes upregulated in HM-1:IMSS compared to UG10.

Gene Name	Accession Number	Fold Change	Function
> **EhRab8B**	EHI_127380	50.4	Intracellular trafficking, secretion, and vesicular transport.
**EhRabF1**	EHI_129740	32.2	Intracellular trafficking and signal transduction mechanisms.
**AIG1 family protein, putative**	EHI_180390	31.2	Bacterial defense mechanisms and stress response.
**EhaRabX13**	EHI_065790	20.9	Intracellular trafficking and signal transduction mechanisms.
**EhRab8A**	EHI_199820	20.4	Intracellular trafficking, secretion, and vesicular transport.
**Ras family protein**	EHI_118280	11.9	Intracellular trafficking and signal transduction mechanisms.
**AIG1 family protein putative**	EHI_109120	11.6	Bacterial defense mechanisms and stress response.
**AIG1 family protein**	EHI_022500	11.5	Bacterial defense mechanisms and stress response.
**Lipase, putative**	EHI_072130	11.0	Secondary metabolites triglyceride biosynthesis, transport, and catabolism.
**Ribosomal protein S30, putative**	EHI_088600	9.8	Translation, ribosomal structure, and biogenesis.
**AIG1 family protein**	EHI_115160	6.4	Bacterial defense mechanisms and stress response.
**Hydrolase, carbon-nitrogen family**	EHI_035680	5.9	Nitrogen compound metabolic process.
**Protein kinase putative**	EHI_023610	4.1	Protein phosphorylation, inorganic ion transport, energy production, and conversion.
**Ran GTPase-activation protein, putative**	EHI_185290	3.0	Regulates nuclear transport and the union and exchange of GTPs.
**EhRabX23**	EHI_107140	2.9	Intracellular trafficking and signal transduction mechanisms.
**Glucosamine-6-phosphate isomerase, putative**	EHI_174640	2.9	Carbohydrate transport and metabolism.
**EhRabC6**	EHI_194280	2.8	Intracellular trafficking, secretion, and vesicular transport.
**Protein kinase putative**	EHI_185230	2.7	Protein phosphorylation, inorganic ion transport, energy production, and conversion.
**Choline/Ethanolamine kinase, putative**	EHI_148580	2.5	Phospholipid metabolism, and catalyzes phosphatidylethanolamine and phosphatidylcholine biosynthesis.
**EhRabD2**	EHI_164900	2.4	Intracellular trafficking, secretion, and vesicular transport.
**SKIP/SNW domain protein**	EHI_199600	2.2	Bifunctional regulator that works in the nucleus as a splicing factor by integrating into the spliceosome. Transcriptional activator by interacting with the Paf1 complex.
**Metal-dependent hydrolase, putative**	EHI_054690	2.2	Hydrolase activity.
**WD domain-containing protein**	EHI_170080	2.1	Assembly of protein complexes or mediators of transient interplay among other proteins.
**tRNA (guanine-N(1)-)-methyltransferase**	EHI_168400	2.1	Biosynthesis, signal transduction, protein repair, chromatin regulation, and gene silencing.
**Ubiquitin ligase, putative**	EHI_104570	2.0	Ubiquitin mediated proteolysis.

**Table 3:** Subset of genes upregulated in UG10 compared to HM-1:IMSS.

Gene Name	Accession Number	Fold Change	Function
**WH2 motif domain-containing protein**	EHI_050810	11.2	Cell contractility, cell motility, cell trafficking, and cell signaling.
**EhRab7G**	EHI_187090	11.1	Intracellular trafficking, secretion, and vesicular transport.
**Heat shock protein 70, putative**	EHI_123490	8.3	Post-translational modification, protein turnover, chaperones.
**Iron-sulfur flavoprotein, putative**	EHI_022600	8.2	Electron transport and response to oxidative stress.
**Iron-sulfur flavoprotein, putative**	EHI_ 181710	6.2	Electron transport and response to oxidative stress.
**Iron-sulfur flavoprotein, putative**	EHI_067720	5.7	Electron transport and response to oxidative stress.
**Iron-sulfur flavoprotein, putative**	EHI_103260	5.4	Electron transport and response to oxidative stress.
**Methionine gamma-lyase, putative**	EHI_057550	5.0	Amino acid transport and metabolism.
**SURFACE antigen ariel1-RELATED**	EHI_172850	4.4	Surface protein important in adhesion and phagocytosis.
**Myb-like DNA-binding domain containing protein**	EHI_166410	4.2	Encodes for nuclear DNA-binding proteins.
**U2 snRNP auxiliary factor, putative**	EHI_192500	3.9	Interaction with the 3’ splice site dinucleotide AG is essential for regulated splicing.
**Surface antigen ariel1, putative**	EHI_080200	3.5	Surface protein important in adhesion and phagocytosis.
**Surface antigen ariel1, putative**	EHI_101730	2.3	Surface protein important in adhesion and phagocytosis.
**Vacuolar protein sorting 35, putative**	EHI_086580	2.1	Intracellular protein transport, lysosome organization, retrograde transport, endosome to Golgi.

**Table 4:** Quantitative expression levels of selected genes for several *Entamoeba histolytica* strains.

		Expression Levels (X-Fold) by qRT-PCR			
Protein Name	Gene Accession	HM-1 ^a^	UG10	G3	Rahman
AIG, putative	EHI_180390	1	−12.7 ± 1.2 (−31.2 ^b^)	−3.4 ± 0.2	ND
EhRab8A	EHI_199820	1	−26.3 ± 1.9 (−20.4 ^b^)	1	1
WH2 motif domain-containing protein	EHI_050810	1	9.6 ± 0.7 (11.2 ^b^)	8.3 ± 0.9	10.5 ± 0.9
Heat shock protein 70, putative	EHI_123490	1	12.1 ± 0.9 (8.3 ^b^)	8.6 ± 0.5	1.55 ± 0.1

^a^ Fold change is relative to HM1 strain expression. ^b^ Fold changes from microarray data. ND: Not detected.

**Table 5:** AIG1 gene expression in monoxenic culture of HM-1 and UG10 strains with *E. coli* O55.

	Strain	
Condition	HM-1	UG10
Axenic	12.7 ± 1.2	1.0
*E. coli* O55 (2 days)	6.86 ± 1.1	2.35 ± 0.2
*E. coli* O55 (30 days)	12.33 ± 1.7	4.13 ± 0.3

The lowest expression of the *EhAIG1* gene without exposure to *E. coli* was in the UG10 strain, and a value of 1 as assigned. Fold change for the other conditions was assigned with respect to the axenic UG10 strain.

## Data Availability

Microarray data are available at the ArrayExpress database hosted at www.ebi.ac.uk/arrayexpress (accessed on 20 January 2023), under the accession number E-MTAB-3525.

## Supplementary Materials

The following supporting information can be downloaded at: https://www.mdpi.com/article/10.3390/pathogens12030489/s1, Figure S1: Heatmap analysis of the up and down-regulated genes comparison between HM1:IMSS and UG10 strains; Figure S2: Virulence of UG10 strain; Figure S3: Topology and structural features of other AIG1 homologs showing high expression in *E. histolytica*; Table S1: Primer used for amplification in qPCR assays; Table S2: Primer used for amplification in endpoint PCR assays; Table S3: Genes upregulated in HM-1:IMSS compared to UG10; Table S4: Genes upregulated in UG10 compared to HM-1:IMSS; Table S5: Quantitative expression levels of classical virulence genes for HM-1 and UG10 *Entamoeba histolytica* strains; Table S6: EHI_180390 gene expression in *E. histolytica* strains.
