# Supplementary material for: Attenuation of In Vitro and In Vivo Virulence Is Associated with Repression of Gene Expression of AIG1 Gene in Entamoeba histolytica
Source: Pathogens. 2023 Mar 21;12(3):489. doi: 10.3390/pathogens12030489 (PMC10051847; doi:10.3390/pathogens12030489)
Supplement: Supplementary file 1 [file pathogens-12-00489-s001.zip › Supplementary material/Figure S1.pdf]

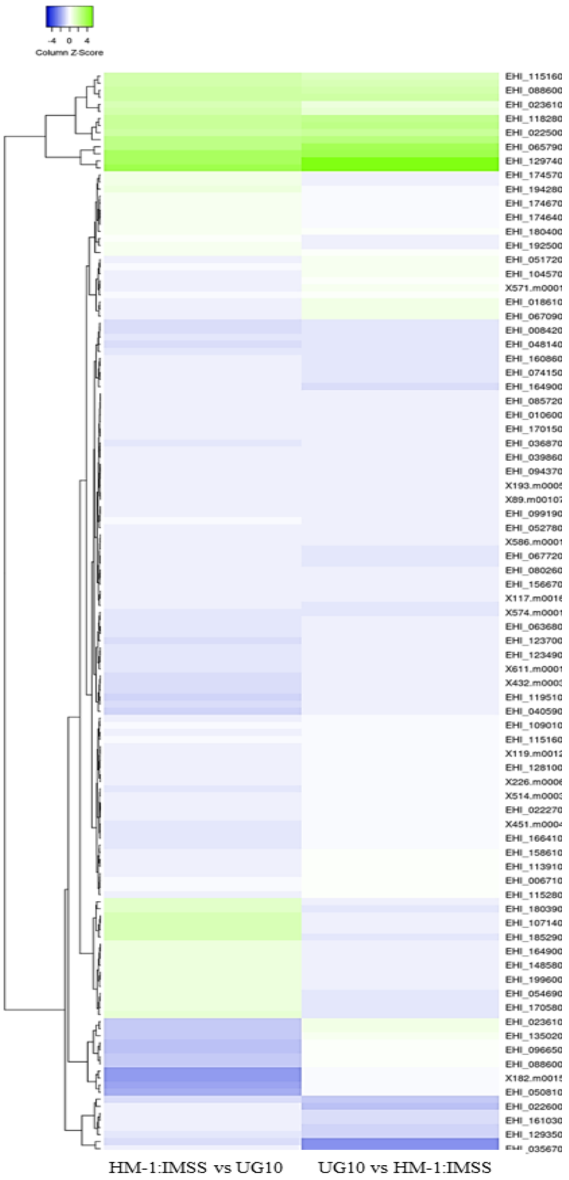

FIG S1. Heatmap analysis of the up and down-regulated genes comparison between HM1:IMSS and UG10 strains. The heatmap was constructed using the most significant up and downregulated genes in the two datasets. Heatmap was generated with Heatmapper (Babicki S, Arndt D, Marcu A, Liang Y, Grant JR, Maciejewski A, Wishart DS. Heatmapper: web-enabled heat mapping for all. Nucleic Acids Res. 2016 Jul 8;44(W1):W147-53. doi: 10.1093/nar/gkw419.). Green indicates downregulated genes and blue, upregulated genes. Parameters were using average linkage with Euclidian distance measurement method.
