# Supplementary material for: Attenuation of In Vitro and In Vivo Virulence Is Associated with Repression of Gene Expression of AIG1 Gene in Entamoeba histolytica
Source: Pathogens. 2023 Mar 21;12(3):489. doi: 10.3390/pathogens12030489 (PMC10051847; doi:10.3390/pathogens12030489)
Supplement: Supplementary file 1 [file pathogens-12-00489-s001.zip › Supplementary material/Figure S2.pdf]

A

G418	Strain	Number of hamsters inoculated/ with abscesses
-	HM-1	5 / 5
+	T10	5 / 5
-	UG10	10 / 0
+	UG10	10 / 0

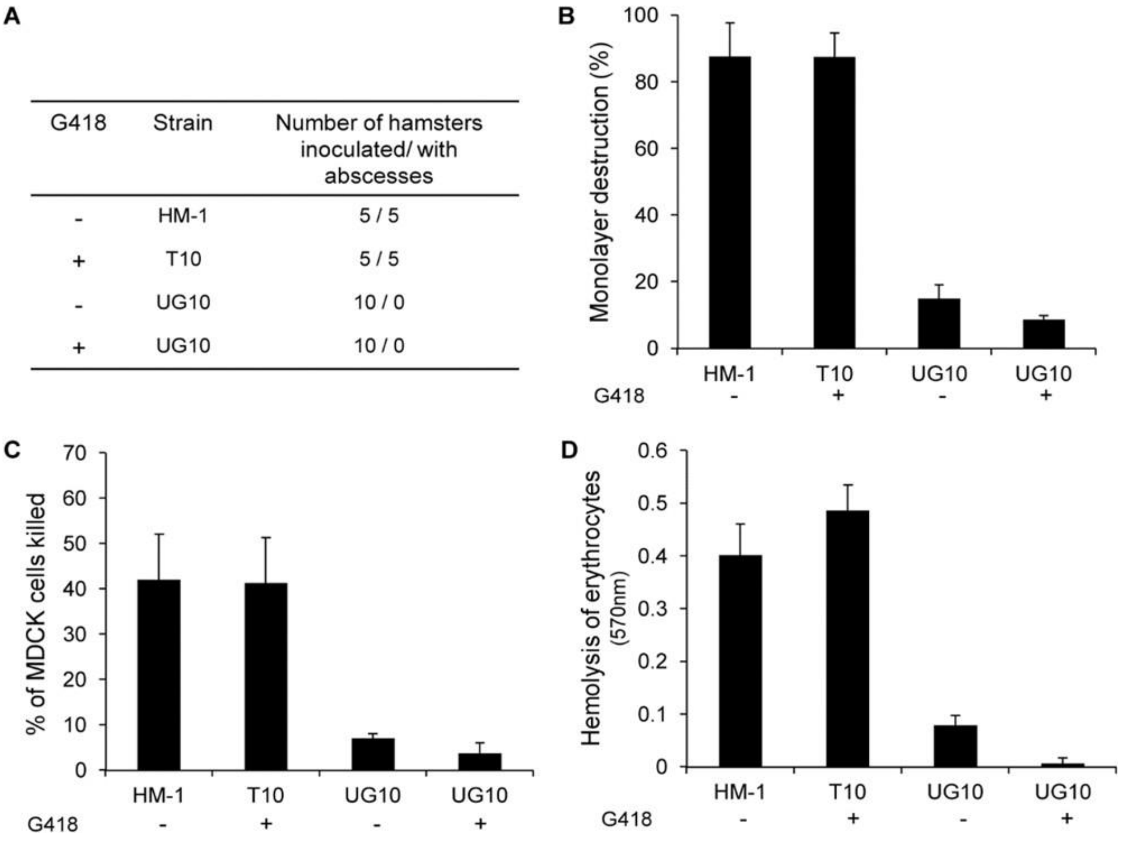

FIG S2. Virulence of UG10 strain. A. Induction of amoebic liver abscesses in hamsters. T10 and UG10 strains were grown with and without 10 μg/ml of G418**.** Hamsters were inoculated intrahepatically with $5x10^5$ trophozoites. HM-1 and T10 induce abscesses affecting 80% of the hepatic lobe. UG10 mutant did not produce abscesses even with $3x10^6$ trophozoites. B. Cytopathic activity. The rate of destruction of MDCK cells monolayer was determined after incubation with $1x10^5$ trophozoites for 60 min at 37°C. C. Cytotoxic activity. Percentage of dead MDCK cells after 60 min of interaction with amoebae was determined by counting fluorescent cells that incorporated the vital dye Hoechst 33258. D. Hemolytic activity. Hemolysis of human erythrocytes by intact trophozoites was determined by spectrophotometric quantification of hemoglobin released after 90 min of interaction at 37°C. Three independent experiments were performed in triplicate for each *in vitro* biological assay.
