# Supplementary material for: Attenuation of In Vitro and In Vivo Virulence Is Associated with Repression of Gene Expression of AIG1 Gene in Entamoeba histolytica
Source: Pathogens. 2023 Mar 21;12(3):489. doi: 10.3390/pathogens12030489 (PMC10051847; doi:10.3390/pathogens12030489)
Supplement: Supplementary file 1 [file pathogens-12-00489-s001.zip › Supplementary material/Table S4.pdf]

Table S4. Genes upregulated in UG10 compared to HM-1:IMSS

Accession number	x-fold change	Gene annotation
EHI_047820	35.470085	Hypothetical protein
EHI_058100	24.578333	Hypothetical protein
EHI_125020	11.964578	Hypothetical protein
EHI_027750	11.405656	Hypothetical protein
EHI_050810	11.189768	WH2 motif domain contaning protein
EHI_187090	11.131988	Rab family GTPase
EHI_178000	10.960888	Hypothetical protein
EHI_050985	10.440165	Hypothetical protein
EHI_065260	10.397165	Hypothetical protein
EHI_107040	10.112236	Hypothetical protein
EHI_039590	9.344586	Hypothetical protein
EHI_149530	8.659974	Hypothetical protein
EHI_134960	8.332713	S-phase kinase-associated protein 1A
EHI_163480	8.294479	Heat shock protein 90, putative
EHI_123490	8.284656	Heat shock protein 70, putative
EHI_022600	8.218277	Iron-sulfur flavoprotein, putative
EHI_193060	8.029789	Hypothetical protein
EHI_103260	7.8080792	Iron-sulfur flavoprotein, putative
EHI_171070	7.7838106	Conservated hypothetical protein
EHI_014290	7.078602	Conservated hypothetical protein
EHI_022270	6.501204	Hypothetical protein
EHI_050490	6.311656	Hypothetical protein
EHI_106290	6.2242885	Hypothetical protein
EHI_194180	6.223766	Hypothetical protein
EHI_181710	6.1745934	Iron-sulfur flavoprotein, putative
EHI_041580	5.8728304	Conservated hypothetical protein
EHI_067720	5.6632223	Iron-sulfur flavoprotein, putative
EHI_201420	5.4009976	Hypothetical protein
EHI_102490	5.1167483	Conservated hypothetical protein
EHI_057550	5.030111	Methionine gamma-lyase, putative
EHI_083760	4.6744676	Conservated hypothetical protein
EHI_114000	4.542217	Hypothetical protein
EHI_083760	4.46397	Hypothetical protein
EHI_172850	4.442909	SURFACE antigen ariel1-RELATED
EHI_166410	4.2159805	Myb-like DNA-binding domain containing protein
EHI_192500	3.896756	U2 snRNP auxiliary factor, putative
EHI_135140	3.7202058	Hypothetical protein
EHI_144390	3.5781713	Hypothetical protein
EHI_080200	3.5284407	Surface antigen ariel1, putative
EHI_123700	3.423494	Hypothetical protein
EHI_083760	3.3183644	Hypothetical protein
EHI_135140	3.3126054	Hypothetical protein
EHI_041580	3.2655358	Conservated hypothetical protein
EHI_027750	3.2400677	Hypothetical protein
EHI_040440	3.2022326	Hypothetical protein

EHI_171070	2.867967	Hypothetical protein
EHI_067090	2.643976	Hypothetical protein
EHI_132420	2.632977	Hypothetical protein
EHI_160850	2.6274683	Hypothetical protein
EHI_160910	2.410206	Hypothetical protein
EHI_101730	2.3067477	Surface antigen ariel1, putative
EHI_086580	2.1185293	Vacuolar protein sorting 35, putative
EHI_036870	2.1124442	Hypothetical protein
EHI_086570	2.0507612	Hypothetical protein
EHI_119510	2.036392	Hypothetical protein
EHI_170590	1.9925152	Hypothetical protein
EHI_166840	1.9784436	Hypothetical protein
EHI_036890	1.9760622	ATPase, Vps4 oligomerisation domain containing protein
EHI_153680	1.9444319	Carbohydrate degrading enzyme, putative
EHI_161030	1.8471569	Rab family GTPase
EHI_156670	1.8327076	Serine/threonine-protein kinase 3, putative
EHI_156310	1.8271518	Ribonuclease, putative
EHI_036900	1.8162141	DEAD/DEAH box helicase, putative
EHI_012990	1.7510566	Hypothetical protein
EHI_160890	1.7475164	Hypothetical protein
EHI_074150	1.7419256	Hypothetical protein
EHI_189970	1.7296472	Amino acid permease, putative
EHI_129350	1.6729357	Hypothetical protein, conserved
EHI_192750	1.6614851	Hypothetical protein, conserved
EHI_041580	1.6552237	Hypothetical protein
EHI_131520	1.6309185	Hypothetical protein
EHI_006710	1.6155498	Hypothetical protein, conserved
EHI_129780	1.5924968	Hypothetical protein
EHI_160860	1.5869534	Inositol polyphosphate 5-phosphatase, putative
EHI_192490	1.5807892	Hypothetical protein
EHI_149840	1.5573486	Hypothetical protein, conserved domain containing
EHI_197410	1.5567738	Cation transporter, putative
EHI_158610	1.5472578	Activator 1 140 kDa subunit, putative
EHI_146980	1.5348405	Hypothetical protein, conserved domain containing
EHI_017660	1.5024832	Hypothetical protein
EHI_113910	1.4979384	Hypothetical protein
EHI_128110	1.4896221	Rab family GTPase
EHI_009800	1.4726119	Phosphatidylserine synthase, putative
EHI_166370	1.4679496	Hypothetical protein
EHI_063680	1.4234157	Hypothetical protein
EHI_128100	1.4137117	Peptidyl-prolyl cis-trans isomerase, putative
EHI_109010	1.4021786	Hypothetical protein
EHI_116470	1.4010212	Hypothetical protein, conserved domain containing
EHI_085720	1.3790215	Hypothetical protein
EHI_161040	1.3732705	Hypothetical protein

EHI_100360	1.3675051	CLL-associated antigen KW-2, putative
EHI_080260	1.3669443	Hypothetical protein
EHI_099190	1.3651347	WD repeat protein
EHI_051720	1.3376491	Transporter, major facilitator family
EHI_076870	1.3344749	Steroid 5-alpha reductase, putative
EHI_167050	1.3182443	60S ribosomal protein L34, putative
EHI_006690	1.3155744	DNA polymerase delta catalytic subunit, putative
EHI_170580	1.3137	Sec61 alpha subunit, putative
EHI_006810	1.3107325	14-3-3 protein 3
EHI_127260	1.3056942	PH domain protein
EHI_029050	1.2891352	Long-chain-fatty-acid--CoA ligase, putative
EHI_099810	1.2834158	Exocyst complex component sec10, putative
EHI_146950	1.2718871	Acid phosphatase, putative
EHI_194060	1.2609698	CAAX amino terminal protease family
EHI_159570	1.2528473	Mob1/phocein family protein
EHI_039860	1.2154683	Hypothetical protein
EHI_080250	1.2091007	Hypothetical protein
EHI_153450	1.1904798	Transporter, major facilitator family
EHI_105140	1.1828128	40S ribosomal protein S15a, putative
EHI_155660	1.1595054	Elongation factor 2, putative
EHI_146180	1.0913334	Rho family GTPase
