# Supplementary material for: Attenuation of In Vitro and In Vivo Virulence Is Associated with Repression of Gene Expression of AIG1 Gene in Entamoeba histolytica
Source: Pathogens. 2023 Mar 21;12(3):489. doi: 10.3390/pathogens12030489 (PMC10051847; doi:10.3390/pathogens12030489)
Supplement: Supplementary file 1 [file pathogens-12-00489-s001.zip › Supplementary material/Table S5.pdf]

Table S5 Quantitative expression levels of classical virulence genes for HM-1 and UG10 *Entamoeba histolytica* strains.

	Strain	
**Gene**	**HM-1**	**UG10**
Eh IgL	1.0	1.60 ± 0.34
Eh IgL	1.0	1.42 ± 0.06
Eh IgL	1.0	1.01 ± 0.24
Eh CP5	1.0	1.21 ± 0.02
Eh adh 112	1.0	1.09 ± 0.07
Ef ap-a	1.0	1.00 ± 0.07
Ef ap-b	1.0	1.45 ± 0.10
Ef ap-c	1.0	0.95 ± 0.04

Data were normalized using the $^{\Delta\Delta}$CT method using the constitutive *Ehgapdh* gene as a control. Gene expression in the control HM-1 strain was assigned a relative value of 1. Gene values for UG10 strain represent expression folds with respect to those in the HM-1 strain.
