# Supplementary material for: Attenuation of In Vitro and In Vivo Virulence Is Associated with Repression of Gene Expression of AIG1 Gene in Entamoeba histolytica
Source: Pathogens. 2023 Mar 21;12(3):489. doi: 10.3390/pathogens12030489 (PMC10051847; doi:10.3390/pathogens12030489)
Supplement: Supplementary file 1 [file pathogens-12-00489-s001.zip › Supplementary material/Table S1.pdf]

**Table S1. Primer used for amplification in qPCR assays.**

| Gene name | Access | Sequence |
|---|---|---|
| **AIG1 family protein** | EHI_180390 (F)* | 5´-TAACGATTCCAAAGGTCAAGAAA-3’ |
| | EHI_180390 (R)* | 5´-AAAAACACATGCACTTTGTCCATACT-3’ |
| **EhRab8A** | EHI_199820 (F) | 5´-TTGTTGGAAATAAGTGTGATG-3’ |
| | EHI_199820 (R) | 5´-GATTCAGTTGGTTGGGTTT-3’ |
| **WH2 motif domain contaning protein** | EHI_050810 (F) | 5´-TACACCACAACCAAAAGACA-3’ |
| | EHI_050810 (R) | 5´-CTTTAACATCAGTGCTTCCA-3’ |
| **Heat shock protein 70** | EHI_123490 (F) | 5´-AACAAAGACAAGTTATTTCACAC-3’ |
| | EHI_123490 (R) | 5´-CCTCCACCAAAATCAAACAC-3’ |
| **Glyceraldehyde-3-phosphate dehydrogenase** | EHI_008200 (F) | 5’-ATTAAAATATGATACCGTCCACAGA-3’ |
| | EHI_008200 (R) | 5’-TTCAACAACATAATCAACTCCAA-3’ |
| **Amoebapore A** | EHI_159480 (F) | 5’-GCAACCTTTGCACTGGACTT-3’ |
| | EHI_159480 (R) | 5’-GCATTGGCATCAACTTTGTC-3’ |
| **Amoebapore B** | EHI_194540 (F) | 5’-TTGATGGTGCTCAAGCTGTT-3’ |
| | EHI_194540 (R) | 5’-CTTTTCGCAAACAACGACTG-3’ |
| **Amoebapore C** | EHI_118270 (F) | 5’-TCCTGTTTGTACATCACTTGTTGG-3’ |
| | EHI_118270 (R) | 5’-GCACAGAGTGTTTCAAGATAATCAGTTAC-3’ |
| **Cysteine proteinase 5** | EHI_168240 (F) | 5’-GCTGCTATGACTGAGGCTGA-3’ |
| | EHI_168240 (R) | 5’-TGATCCACATGATGCTTGGT-3’ |
| **Gal-lectin 35kDa** | EHI_035690 (F) | 5’-TGCACATGTCCAATGTGTTG-3’ |
| | EHI_035690 (R) | 5’-CCATGCATTTCACTTTTGGTT-3’ |
| **Gal-lectin subunit Igl2** | EHI_065330 (F) | 5’-GCCAGAAAAGCAACTGATGGT-3’ |
| | EHI_065330 (R) | 5’-TGCTTCAGTTGCACTACACC-3’ |
| **Gal-lectin light subunit** | EHI_148790 (F) | 5’-GCACTAAAAACTCCACTCTCAGC-3’ |
| | EHI_148790 (R) | 5’-TTGCTGATACGTCTGATGGAC-3’ |
| **Glyceraldehyde-3-phosphate dehydrogenase** | EHI_008200 (F) | 5’-ATTAAAATATGATACCGTCCACAGA-3’ |
| | EHI_008200 (R) | 5’-TGAGTAGCAGTAGTAGCATGAATAGTT-3’ |

* F = Forward

*R = Reverse
