# Supplementary material for: Attenuation of In Vitro and In Vivo Virulence Is Associated with Repression of Gene Expression of AIG1 Gene in Entamoeba histolytica
Source: Pathogens. 2023 Mar 21;12(3):489. doi: 10.3390/pathogens12030489 (PMC10051847; doi:10.3390/pathogens12030489)
Supplement: Supplementary file 1 [file pathogens-12-00489-s001.zip › Supplementary material/Table S2.pdf]

Table S2. Primer used for amplification in endpoint PCR assays.

Gene name	Access	Sequence
**P-glycoprotein family protein 1**	EHI_114150 (F)*	5’-AACAGTGCTACAACATCCTT-3’
	EHI_114150 (R)*	5’-TCATGTGCATTTGCCATTTTA-3’
**P-glycoprotein family protein 5**	EHI_175450 (F)	5’-GTAGGAGGTGCAGTATTTCC-3’
	EHI_175450 (R)	5’-CCATCCTATTTCTTGTTTGAC-3
**AIG1 protein**	EHI_180390 (F)	5’-TAACGATTCCAAAGGTCAAGAAA-3’
	EHI_180390 (R)	5’-AAAAACACATGCACTTTGTCCATACT-3’
**EhRab8A**	EHI_199820 (F)	5’-ATTGGAGAAAGTGGAGTTGGGAAAA-3’
	EHI_199820 (R)	5’-AGTTGATTCAGTTGGTTGGGTTTGT-3’
**WH2 motif domain contaning protein**	EHI_050810 (F)	5’-TACACCACAACCAAAAGACA-3’
	EHI_050810 (R)	5’-CTTTAACATCAGTGCTTCCA-3’
**Heat shock protein 70**	EHI_123490 (F)	5’-AACCAACTGCAGCCGCATTATATTA-3’
	EHI_123490 (R)	5’-TACAGGAACAATTGCAAAACCACGA-3’
**Amoebapore A**	EHI_159480 (F)	5’-CAAGCATGAATCTTAGCAC-3’
	EHI_159480 (R)	5’-CTACTCACCAAGGAGAAATC-3’
**Gal-lectin 35kDa**	EHI_035690 (F)	5’-CATTTGGTAAGACTCAAGACGGA-3’
	EHI_035690 (R)	5’-TGGTGCATCTGATAATGGTGGGTT-3’
**Gal-lectin lectin light subunit**	EHI_148790 (F)	5’-ATTGCTGATACGTCTGATGGAC-3’
	EHI_148790 (R)	5’-GTTCCTGGGTCTAATGGTGCTT-3’
**Gal-lectin Igl2**	EHI_065330 (F)	5’-TTCTGTAGACATTAAACCACCACCA-3’
	EHI_065330 (R)	5’-ATTCTTCTACTATTTTCTTCTTCTCA-3’
**Cysteine proteinase 5**	EHI_168240 (F)	5’-AAAGAATGTTCATCAACTCAGCTT-3’
	EHI_168240 (R)	5’-TTAAGCATCAGCAACCCCAACTGG-3’
**Small subunit ribosomal RNA gene**	ssr RNA (F)	5’-GTACAAAATGGCCAATTCATTCAATG-3’
	ssr RNA (R)	5’-ACTACCAACTGATTGATAGATCAG-3’
**Neomycin resistance family**	pEhEx plasmid (F)	5’-ATGATTGAACAAGATGGATTGCAC-3’
	pEhEx plasmid (R)	5’-TCAGAAGAACTCGTCAAGAAGGC-3’
**AIG1 protein (overexpressing)**	EHI_180390 (F)	5’-GGAAGATCTATGAAACAAACTAAGTTAC-3’
	EHI_180390 (R)	5’- CCGCTCGAGTTAATTTGACCACTT-3’

* F = Forward
*R = Reverse
