# Supplementary material for: Attenuation of In Vitro and In Vivo Virulence Is Associated with Repression of Gene Expression of AIG1 Gene in Entamoeba histolytica
Source: Pathogens. 2023 Mar 21;12(3):489. doi: 10.3390/pathogens12030489 (PMC10051847; doi:10.3390/pathogens12030489)
Supplement: Supplementary file 1 [file pathogens-12-00489-s001.zip › Supplementary material/Table S3.pdf]

Table S3. Genes upregulated in HM-1:IMSS compared to UG10

Accession number	x-fold change	Gene annotation
EHI_035670	155.9296	Hypothetical protein
EHI_042720	51.956474	60S ribosomal protein L29
EHI_127380	50.387486	Rab family GTPase
EHI_022490	46.480103	AIG1 family protein, putative
EHI_129740	32.194775	Rab family GTPase
EHI_180390	31.23464	AIG1 family protein, putative
EHI_038440	25.656067	Hypothetical protein
EHI_065790	20.877924	Rab family GTPase
EHI_199820	20.453114	Rab family GTPase
EHI_041465	13.361166	AIG1 family protein, putative
EHI_118280	11.861932	Ras family protein
EHI_109120	11.595099	AIG1 family protein, putative
EHI_022500	11.464334	AIG1 family protein
EHI_072130	11.015177	Lipase, putative
EHI_088600	9.8585205	Ribosomal protein S30, putative
EHI_142160	8.95683	Hypothetical protein
EHI_115160	6.455674	AIG1 family protein
EHI_127440	6.3614845	Tyrosine kinase, putative
EHI_035680	5.8673716	Hydrolase, carbon-nitrogen family
EHI_163480	5.4945803	90 kDa heat shock protein, putative
EHI_023620	5.1501603	Hypothetical protein
EHI_094370	4.851954	Hypothetical protein
EHI_174570	4.5171866	Hypothetical protein
EHI_136940	4.451498	AIG1 family protein, putative
EHI_176580	4.394288	AIG1 family protein, putative
EHI_185250	4.327921	Hypothetical protein
EHI_174600	4.2813544	Hypothetical protein
EHI_023610	4.1226306	Protein kinase, putative
EHI_180400	3.37778	Hypothetical protein
EHI_185230	3.2681186	Protein kinase, putative
EHI_185260	3.2194395	Hypothetical protein
EHI_185290	3.002424	Ran GTPase-activating protein, putative
EHI_107140	2.9975307	Rab family GTPase
EHI_174640	2.97003	Glucosamine-6-phosphate isomerase, putative
EHI_194280	2.806804	Rab family GTPase
EHI_185230	2.7505772	Protein kinase, putative
EHI_092310	2.5931044	Hypothetical protein
EHI_144390	2.5532148	Hypothetical protein
EHI_148580	2.5328627	Choline/ethanolamine kinase, putative
EHI_174510	2.486747	Hypothetical protein

EHI_174580	2.4361563	Hypothetical protein
EHI_164900	2.3615165	Rab family GTPase
EHI_114770	2.3058298	Hypothetical protein
EHI_199600	2.2366023	SKIP/SNW domain protein
EHI_054690	2.1680312	Metal dependent hydrolase, putative
EHI_170080	2.1210775	WD domain containing protein
EHI_187080	2.0843754	Hypothetical protein
EHI_168400	2.0842412	tRNA (guanine-N(1)-)-methyltransferase TRM10
EHI_151930	2.0600083	hypothetical protein, conserved
EHI_104570	2.0508208	ubiquitin ligase, putative
EHI_048190	2.0240479	Hypothetical protein
EHI_154690	1.8927152	Hypothetical protein
EHI_098250	1.8904233	MutT/nudix family protein
EHI_048140	1.8661666	Hypothetical protein
EHI_120930	1.8553259	Protein kinase domain containing protein
EHI_100260	1.8314421	Hypothetical protein, conserved
EHI_008420	1.8193461	Hypothetical protein
EHI_055340	1.8114331	Rho GTPase activating protein
EHI_170150	1.8038363	Hypothetical protein
EHI_199620	1.7798458	Hypothetical protein, conserved
EHI_185280	1.7648082	Phosphoglucomutase/phosphomannomutase family protein
EHI_008680	1.7334363	Ubiquitin carboxyl-terminal hydrolase family protein
EHI_170020	1.6425189	Beta-galactosidase, putative
EHI_050650	1.6322083	Protein kinase, putative
EHI_105280	1.628754	Hypothetical protein
EHI_073350	1.6281565	HAD hydrolase, family IA, variant 3
EHI_124420	1.6024048	Hypothetical protein
EHI_115280	1.6017302	Hypothetical protein
EHI_021190	1.5774556	Hypothetical protein
EHI_180780	1.560256	Hypothetical protein
EHI_186990	1.5377846	Tyrosine kinase, putative
EHI_087640	1.5316033	Protein kinase with ankyrin repeats, putative
EHI_008370	1.5200183	Hypothetical protein
EHI_040470	1.4708986	Hypothetical protein, conserved domain containing
EHI_008610	1.4694138	Hypothetical protein, conserved
EHI_098270	1.4673413	RhoGAP domain containing protein
EHI_018610	1.4464959	Hypothetical protein
EHI_050740	1.4409083	L-asparaginase, putative
EHI_174670	1.4203801	Proteasome beta subunit, putative
EHI_140760	1.4020662	Hypothetical protein

EHI_114780	1.3803723	Hypothetical protein
EHI_088060	1.3618989	Hypothetical protein
EHI_135020	1.3577846	Hypothetical protein
EHI_186950	1.3559422	Protein phosphatase domain-containing protein
EHI_158150	1.3225229	LIM zinc finger domain containing protein
EHI_186770	1.3183279	Actin-binding protein, cofilin/tropomyosin family
EHI_010600	1.3011776	BAR/SH3 domain containing protein
EHI_096240	1.2941746	Hypothetical protein
EHI_052780	1.2731665	TolA-like protein, putative
EHI_148170	1.2597117	Protein phosphatase regulatory subunit, putative
EHI_040590	1.258059	Hypothetical protein
EHI_174140	1.2565365	Hypothetical protein
EHI_096650	1.1870943	Histone H3, putative
