# Supplementary material for: Attenuation of In Vitro and In Vivo Virulence Is Associated with Repression of Gene Expression of AIG1 Gene in Entamoeba histolytica
Source: Pathogens. 2023 Mar 21;12(3):489. doi: 10.3390/pathogens12030489 (PMC10051847; doi:10.3390/pathogens12030489)
Supplement: Supplementary file 1 [file pathogens-12-00489-s001.zip › Supplementary material/Table S6.pdf]

Table S6. EHI_180390 gene expression in *E. histolytica* strains.

Strain	Fold change
UG10	1
UG10spAIG1	6.95±0.9
HM-1	12.7±1.2

The lowest expression of the *EhAIG1* gene was in the UG10 strain, and a value of 1 was assigned. Fold Change for the other strains was assigned with respect to the UG10 strain.
