# Supplementary figures and images for: Attenuation of In Vitro and In Vivo Virulence Is Associated with Repression of Gene Expression of AIG1 Gene in Entamoeba histolytica
Source: Pathogens. 2023 Mar 21;12(3):489. doi: 10.3390/pathogens12030489 (PMC10051847; doi:10.3390/pathogens12030489)

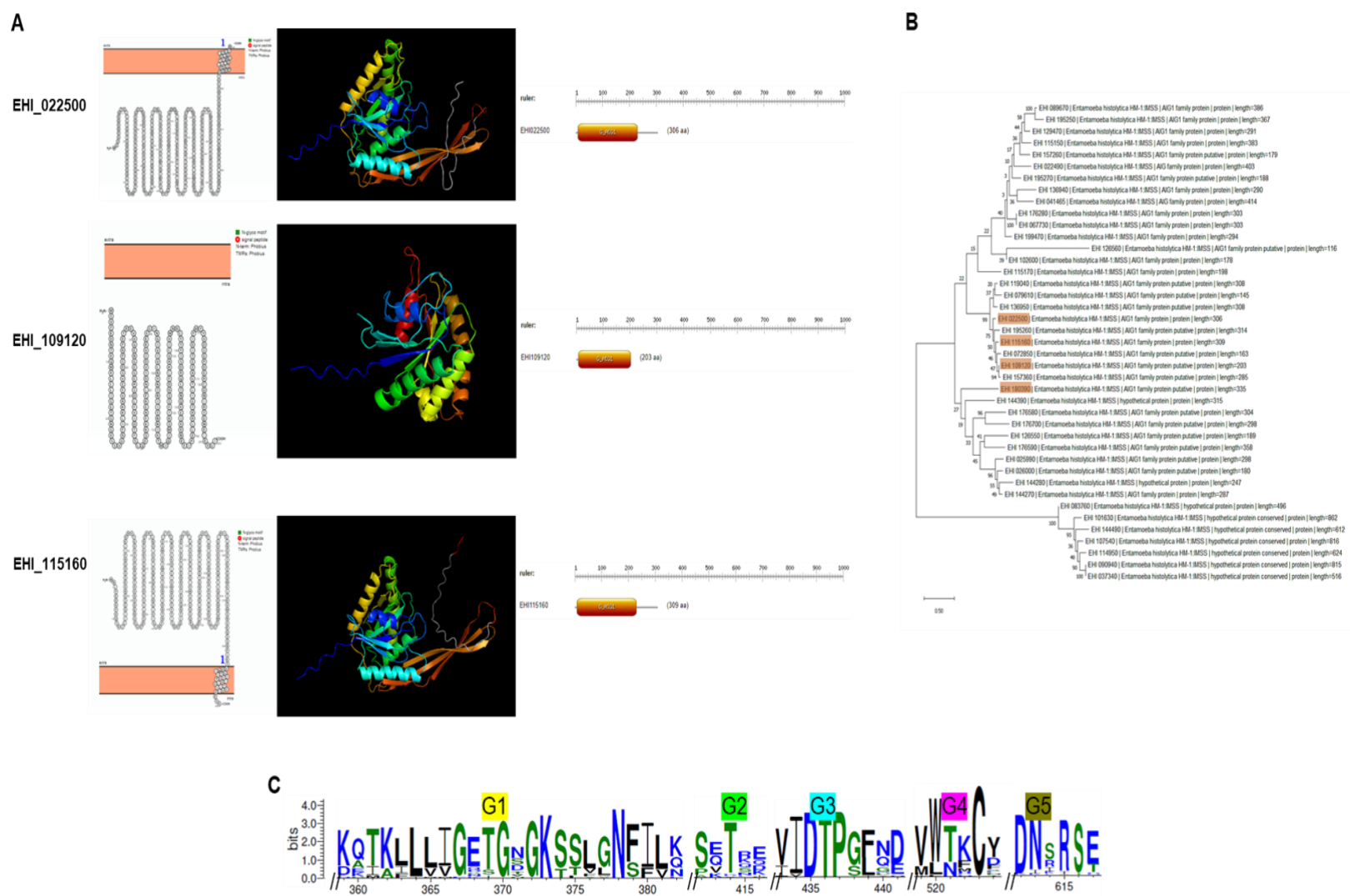

Supplement: Supplementary file 1 [file pathogens-12-00489-s001.zip › Supplementary material/Figure S3.pdf]
